# Improving survival of stage II‐III primary gastric signet ring cell carcinoma by adjuvant chemoradiotherapy

**DOI:** 10.1002/cam4.3342

**Published:** 2020-08-03

**Authors:** Yang Li, Zhikai Zhu, Fuhai Ma, Liyan Xue, Yantao Tian

**Affiliations:** ^1^ Department of Pancreatic and Gastric Surgery National Cancer Center/National Clinical Research Center for Cancer/Cancer Hospital Chinese Academy of Medical Sciences & Peking Union Medical College Beijing China; ^2^ School of Public Health Chinese Academy of Medical Sciences & Peking Union Medical College Beijing China; ^3^ Department of Oncology Georgetown University School of Medicine Washington DC USA; ^4^ Department of Pathology National Cancer Center/Cancer Hospital Chinese Academy of Medical Sciences & Peking Union Medical College Beijing China

**Keywords:** cancer survival, chemoradiotherapy, gastric carcinoma, signet ring cell, treatments

## Abstract

**Background:**

There is no consistent evidence about the appropriate treatment strategies for gastric signet ring cell carcinoma (GSRC) to improve prognosis. We conducted a population‐based study to examine the effects of combined modality therapies on survival outcomes using the Surveillance, Epidemiology, and End Results (SEER) data.

**Methods:**

Analyses included stage II‐III primary GSRC patients who were diagnosed between 2006 and 2016. Therapies were categorized as gastrectomy group, adjuvant chemotherapy (CT) group, neoadjuvant radiotherapy (RT) group, and adjuvant chemoradiotherapy (CRT) group. Survival analyses were conducted by Kaplan‐Meier method and Cox proportional hazards models and subgrouped by gender, tumor site, stage at diagnosis, and number of lymph nodes removed.

**Results:**

Of the 1717 cases of stage II‐III primary GSRC, the mean (SD) age was 59.6 (13.3) years, and over a half were male (52.8%). A total of 39.9% patients received adjuvant CRT and the 5‐year overall survival (OS) rate was 34.6%. The median OS of patients treated with adjuvant CRT was significantly longer than that of the gastrectomy group (33 months vs 24 months, aHR = 0.71, 95% CI: 0.59, 0.84). Although the crude model showed a significant association between adjuvant CT and total survival (cHR = 0.81, 95% CI: 0.68, 0.96), the effect measure turned null in the multivariable and sub‐group analysis. We did not find the significant effect of neoadjuvant RT.

**Conclusions:**

In this study, GSRC patients with stage II‐III experienced improved overall survival after receiving adjuvant CRT, which provides several treatment implications. More clinical trials will be needed to verify the conclusion derived from this study.

## Graphical abstract

The adjuvant CRT can improve overall survival of patients with primary locally advanced GSRC, and the effect of adjuvant CT and neoadjuvant RT was not found.

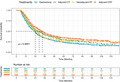

## 1 INTRODUCTION

Gastric cancer (GC) represents the 5th most common cancer worldwide, with an estimated 1 000 000 new cases in 2018, and it is the 3rd leading cause of death by cancer over the world, with approximately 783 000 (8.8%) deaths annually.^1^ Gastric signet‐ring cell carcinoma (GSRC) is a rare subtype of gastric cancer featured by poorly cohesive cells with no gland formation, low differentiation,^2^ and more than 50% poorly cohesive cells having signet ring cell morphology according to the WHO classification 2010.^3^, ^4^ The incidence rate of GSRC in the United States has gradually increased from 0.3 cases per 100 000 persons in 1973 to 1.8 cases per 100 000 persons in 2000.^5^ The prognosis of GSRC was reported to better than that of other gastric adenocarcinomas in I stage,^6^ while the prognosis usually turned to be much poorer in the advanced stage, and 5‐year overall survival (OS) rate was only 0%‐25%.^7^ This high magnificence and poor prognosis feature highlights the importance of effective clinical treatment modalities for GSRC patients.

Current gastric cancer treatment guideline has shown that combined modality therapy (CMT) could significantly increase survival in gastric cancer patients, and postoperative chemoradiotherapy (CRT) or perioperative chemotherapy (CT) are the preferred approaches for treatment of localized gastric cancer.^8^ However, CMT may not be appropriate for GSRC as it is generally found to be resistant to CT or RT. Previous studies found that presence of signet ring cell was associated with a lower rate of pathologic complete response to CRT. It has also been reported that higher fraction of signet ring cell histology is associated with higher chemotherapy resistance.^9^

So far, there is no specific and well‐defined standard of treatment for GSRC. Some studies have shown that CMT provides no survival benefit to patients with GSRC. For example, a multicenter comparative study found that the median survival was shorter in the CRT group than surgery alone group (12.8 months vs 14.0 months). ^10^However, other studies demonstrate a survival advantage for GSRC patients by perioperative CMT. The neoadjuvant CRT group was noted to have a better 3‐year overall survival than surgery alone group (51% vs 21%) among 97 patients with GSRC from 21 French centers.^11^ A study of 310 esophagogastric GSRC patients found that neoadjuvant treatment showed a better median survival time (28.5 months vs 14.9 months).^12^Given the controversial evidence regarding the effect of CMT on survival of GSRC patients, we decided to explore the clinical proper treatment strategies for locally advanced primary GSRC patients using the 2006‐2016 Surveillance, Epidemiology, and End Results (SEER) data.

## 2 MATERIALS AND METHODS

### 2.1 Data source

The National Cancer Institute's SEER program is a nationally representative population‐based cancer reporting system originated in 1974 with seven cancer registries and has grown to include 21 cancer registries in 2016, covering approximately 34.6% of the US population. SEER provides cancer statistics information in an effort to reduce the cancer burden and can be used to conduct researches on cancer incidence, prevalence, and survival.^13^ Detailed information about SEER can be found elsewhere.

### 2.2 Study population

**FIGURE 1:**
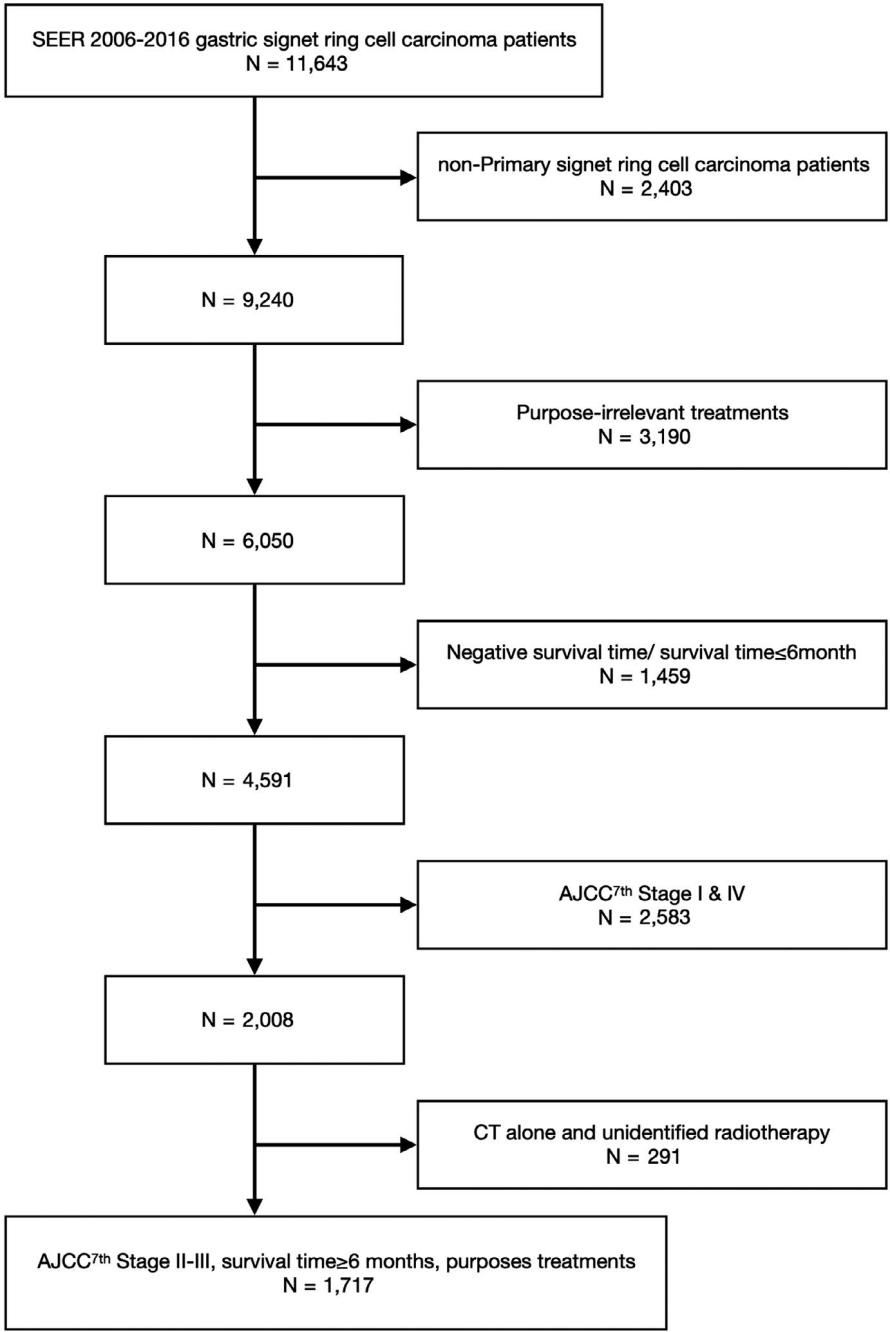
Flow chart

We retrieved the GSRC incident cases and their corresponding demographic and cancer characteristics using SEER*Stat version 8.3.6 software.^14^ The International Classification of Disease 3rd edition (ICD‐O‐3) was used to identify gastric cancer using site codes C160‐6, C168‐9, and histology codes 8940 was used to identify the specific patients with GSRC. Given the therapy period needed for various regimens,^15^ this study excluded patients with survival time less than 6 months. Participants were uniformly reviewed and restaged according to the 7th edition of the American Joint Committee on Cancer Staging Manual (AJCC).^16^ After the further exclusions, a total of 1717 patients with locally advanced primary GSRC from SEER between January 1, 2006 and December 31, 2016 were included for current analysis (Figure 1).

### 2.3 Definition of variables

Treatment was recategorized into four groups: gastrectomy group, adjuvant CT group (gastrectomy plus adjuvant CT), neoadjuvant RT group (gastrectomy with neoadjuvant RT combined adjuvant CT), and adjuvant CRT group (gastrectomy with adjuvant RT combined adjuvant CT). The latter three treatments were considered as CMT in our study.

Follow‐up time in person‐years was used as the time metric and survival was calculated in months from the year of diagnosis to the date of confirmed death, the date they moved out of catchment area, or the end of the follow‐up period (December 31, 2016), whichever occurred first. Outcome was defined as overall survival and GSRC‐specific survival.

Demographic characteristics included age, gender, race, and marital status. Age was treated as an ordinal variable: young (≤44 years), middle‐aged (45‐59 years), and elderly (≥60). Race was categorized as White, Asian/Pacific Islander, Black, and others. Marital status was classified as married and not married (including never married, divorced, widowed, or separated) because those classified as married could receive support from their spouses and have healthier lifestyle that impacted survival.^17^ Tumor site was divided by upper (cardia, fundus, gastroesophageal junction), middle (body, lesser/greater curvature), and lower (antrum, pylorus) part of stomach. The AJCC guidelines recommend a minimum of 16 removed lymph nodes or D2 lymphadenectomy for adequate staging; thus, our cut‐off values of lymph nodes removal were set to be 16 and 30 (details were included in Figure S1).^18^ The cut‐off point of lymph nodes removed was set at 20, which can be enough to evaluate prognosis of GSRC patients.^19^

All demographic and clinicopathological factors were selected based on previously published articles^20^, ^21^ and a *prior* knowledge regarding the classification. No statistical method was used to handle missing data.

### 2.4 Statistical analysis

Frequency and proportions were calculated for all demographic and clinic categorical variables. Log‐rank tests were performed to examine if the OS differed by demographic clinicopathological characteristics, and treatment modalities.

When the proportional hazards assumption was held, we used Cox proportional hazards regression to calculate the crude and adjusted hazard ratios (HR) and 95% confidence intervals (CI) for the effect of treatments on survival outcome; the model was adjusted for following potential confounders: age groups, gender, race, marital status, tumor site, histology differentiation, stage at diagnosis, and lymph nodes removed. Several factors, including gender, and clinicopathological factors, could substantially affect patients’ prognosis. Thus, subgroup analysis and interaction tests were conducted by gender, tumor site, stage at diagnosis, and lymph nodes removed to explore if the impact of CMT is stronger in certain groups, allowing them to establish a more targeted medical treatment strategy. Histology differentiation was not considered due to the uneven distribution within the variable.

A *P*‐value <.05 was considered significant for all comparisons for two‐sided test. All statistical analyses were performed with Stata 15.0 (StataCorp, LLP). Figures were produced using the R Survival and Survminer package (version 3.6.1).

## 3 RESULTS

### 3.1 Descriptive statistics

In this study, total of 4574 person‐years of follow‐up accrued over a median of 4.9 years (interquartile range, 4.7‐5.3 years) of observation and GSRC was the predominate cause of mortality in this population, accounting for 89.0% of the overall causes of mortality.

Table 1 presents the overall distribution of demographic, clinicopathologic characteristics, and treatment modalities within the included study population. The mean (SD) age was 59.6 (13.3) years. The majority of the population were male (52.8%), and non‐Hispanic White (66.0%). Patients included in the analysis were generally married (61.9%). Tumor localization were relatively evenly distributed (upper: 30.2%, middle: 28.2%, and lower: 30.3%). Most patients had a poorly differentiated or undifferentiated tumor (91.5%) and are in locally advanced stage (stage II: 32.8, stage III: 62.9%). About one‐third of patients had gastrectomy with other organs (32.2%). Half of the patients received radiotherapy (50.0%), and most received chemotherapy (81.1%). Less than one‐fifth (18.2%) of participants had complete D2 lymphadenectomy (≥30 lymph nodes harvesting).

**TABLE 1:** Characteristics of 1717 Stage II‐III GSRC Survivors From 2006 to 2016 SEER

Characteristics	Overall^a^	Person‐year	All causes Mortality^b^	p‐value^c^
	(N = 1717)	(n = 4574)	(n = 1045)	
Age (year)				
≤44	612 (35.6)	1751	351 (57.4)	**<0.01**
45‐59	680 (39.6)	1855	399 (58.7)	
≥60	425 (24.8)	969	295 (69.4)	
Gender				
Male	906 (52.8)	2452	537 (59.3)	0.16
Female	811 (47.2)	2122	508 (62.6)	
Race				
White	1133 (66.0)	2975	693 (61.2)	**0.03**
Asian/Pacific Islander	329 (19.2)	960	182 (55.3)	
Black	229 (13.3)	581	154 (67.3)	
Others	26 (1.5)	58	16 (61.5)	
Marital status				
Not married	655 (38.2)	1565	416 (63.5)	**<0.01**
Married	1062 (61.9)	3010	629 (59.2)	
Tumor site				
Upper	519 (30.2)	1287	327 (63.0)	**<0.01**
Middle	484 (28.2)	1393	269 (55.6)	
Lower	521 (30.3)	1452	316 (60.7)	
NOS^d^	193 (11.2)	444	133 (68.9)	
Histology differentiation				
Well/ moderately differentiated	33 (1.9)	84	19 (58.6)	0.74
Poorly/ un‐differentiated	1571 (91.5)	4166	958 (61.0)	
Unknown	113 (6.6)	325	68 (60.2)	
Stage at diagnosis				
II	563 (32.8)	1865	268 (47.6)	**<0.01**
III	1081 (62.9)	2521	739 (68.4)	
IIIa	471 (27.4)	1317	312 (66.2)	
IIIb	323 (18.8)	723	218 (67.5)	
IIIc	287 (16.7)	481	209 (72.8)	
Unknown	73 (4.3)	188	38 (52.1)	
Treatment of surgery				
Gastrectomy	360 (21.0)	1033	203 (56.4)	**<0.01**
Proximal gastrectomy	391 (22.8)	1122	217 (55.5)	
Distal gastrectomy	56 (3.3)	176	29 (51.8)	
Near‐total or total gastrectomy	358 (20.9)	846	236 (65.9)	
Gastrectomy with other organs	552 (32.2)	1397	360 (65.2)	
Radiotherapy				
No radiotherapy	859 (50.0)	2121	537 (62.5)	**<0.01**
Neoadjuvant radiotherapy	173 (10.1)	368	101 (58.4)	
Adjuvant radiotherapy	685 (39.9)	2086	407 (59.4)	
Chemotherapy				
No Chemotherapy	325 (18.9)	830	234 (72.0)	**<0.01**
Chemotherapy	1392 (81.1)	3745	811 (58.3)	
Lymph nodes removed				
<16	709 (41.3)	1903	484 (68.3)	**<0.01**
≥16 to <30	663 (38.6)	1743	375 (56.6)	
≥30	312 (18.2)	832	167 (53.5)	
Uncertain	33 (1.9)	97	19 (57.6)	

Note

Boldface indicates statistical significance (*P* < .05).

^a^ Column percentage was reported for the overall sample; percentage can differ slightly from 100% because of rounding.

^b^ Row percentage was reported for all‐causes of mortality subpopulation.

^c^ Log‐rank tests were used to compare whether the survival was statistically significant in different risk groups.

^d^ Respondents whose information were not documented were defined as NOS.

Our current study had enough person‐years of follow‐up to investigate possible factors influencing the effect of treatments. Log‐rank tests indicated that patients who were younger, male, Asian/ Pacific Islander, married, middle tumor site, well/ moderately differentiated, earlier stage at diagnosis, received CRT or ≥30 lymph nodes removed were more likely to have a better survival (*P*s < .05).

### 3.2 Survival analysis

**FIGURE 2:**
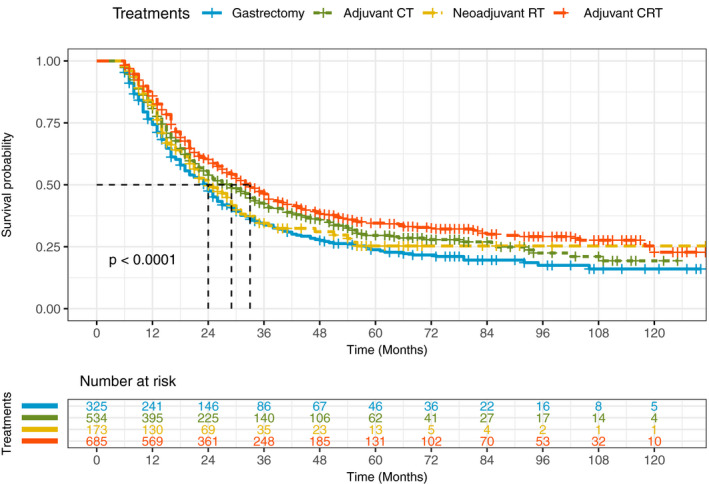
Adjusted overall survival curves for GSRC patients who received combined modality therapy (CMT) compared with those who received gastrectomy alone patients had a median follow‐up of 21.0 months (interquartile range, 13.0‐42.0 months)

**FIGURE 3:**
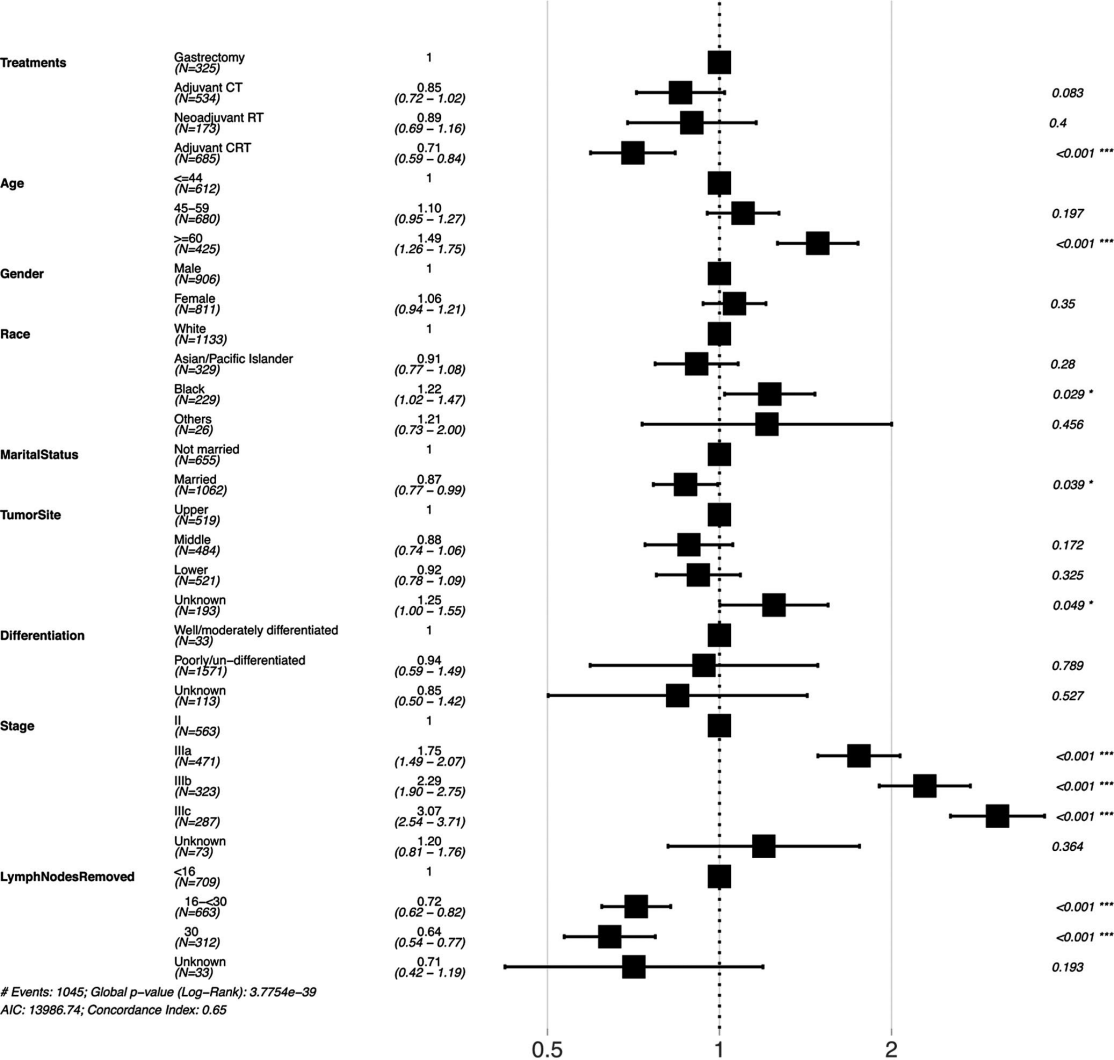
Forest plot of prognosis factors for GSRC overall survival

The Cox proportional hazards regression model depicted in Table 2, showed that 18.9% of the patients underwent gastrectomy alone and 81.1% patients received CMT (adjuvant CT group, 31.1%; neoadjuvant RT group, 10.1%; adjuvant CRT group, 39.9%). The overall 5‐year survival rate was 23.8% for only gastrectomy and 29.6%, 25.3%, and 34.6% for combined therapy respectively. In unadjusted Cox proportional hazards regression analyses, use of adjuvant CRT was associated with improvement in OS (cHR = 0.70, 95% CI: 0.59, 0.82).When adjusted for other variables, the association remained robust for total mortality (aHR = 0.71, 95% CI: 0.59, 0.84) and GSRC specific mortality (aHR = 0.75, 95% CI: 0.63, 0.91). We also found potentially positive associations between adjuvant CT group and overall survival in crude model (cHR = 0.81, 95% CI = 0.68, 0.96), however, effect measures were not statistically significant after the covariates were included (aHR = 0.85, 95% CI: 0.72, 1.02). There is no significant survival benefit from neoadjuvant RT group compared to gastrectomy group (aHR = 0.89, 95% CI: 0.69, 1.16). Figure 2 survival curves shows survival probability for GSRC patients underwent gastrectomy alone, CMT, and the number of patients at risk. The median overall survival of adjuvant CRT group was significantly longer than that of the gastrectomy group patients (33.0 months vs 24.0 months). Figure 3 forest plot showed that being younger (HR_≥60 vs ≤44_ = 1.49, 95% CI: 1.26, 1.75), earlier AJCC stage at diagnosis (HR_IIIc vs II_ = 3.07, 95% CI: 2.54, 3.71), or more lymph nodes removed (HR_≥30 vs <16_ = 0.64, 95% CI: 0.54, 0.77) were significantly associated with improved overall survival.

**TABLE 2:** Risk of mortality according to the treatments among GSRC survivors from a cox multivariate analysis

Variable	Overall (N = 1717)	5‐year OS rate (%)	cHR (95% CI)	aHR (95% CI)^a^	aHR (95% CI)^b^
Treatments		30.1			
Gastrectomy group (ref)	325 (18.9)	23.8	1.00	1.00	1.00
Adjuvant CT group	534 (31.1)	29.6	**0.81 (0.68, 0.96)**	0.85 (0.72, 1.02)	0.93 (0.77, 1.13)
Neoadjuvant RT group	173 (10.1)	25.3	0.90 (0.71, 1.14)	0.89 (0.68, 1.16)	0.98 (0.75, 1.29)
Adjuvant CRT group	685 (39.9)	34.6	**0.70 (0.59, 0.82)**	**0.71 (0.59, 0.84)**	**0.75 (0.63, 0.91)**

Note

Boldface indicates statistical significance (*P* < .05).

Abbreviations: aHR, adjusted harzard ratio; cHR, crude harzard ratio.

^a^ All causes of mortality was defined as the primary endpoint in the model.

^b^ GSRC specific mortality was defined as the secondary endpoint.

### 3.3 Results of sensitivity analysis

Considering the different potential effects of subgroups defined by gender, tumor site, stage at diagnosis, and lymph nodes removed, we further investigated the effect and observed that the lower risk of mortality was specifically robust in adjuvant CRT group, in comparison with those of gastrectomy group (Table 3). In general, the effect of CMT on survival were observed minimal differences in subgroup. We observed that the aHRs of treatment modalities among each gender group were similar to those in primary multivariable analysis. The results indicated that the positive impact of adjuvant CRT on survival was inconsiderable among patients with stage IIIb (aHR = 0.72, 95% CI: 0.49, 1.05), ≥16 to <30 lymph nodes removed (aHR = 0.83, 95% CI: 0.62, 1.11), ≥30 lymph nodes removed (aHR = 0.63, 95% CI: 0.40, 1.01) or upper tumor site (aHR = 0.89, 95% CI: 0.61, 1.28).

**TABLE 3:** Associations of Treatment and Risk of Mortality Among GSRC Survivors By subgroups

Variable	*Gastrectomy group (ref)*		*Adjuvant CT group*		*Neoadjuvant RT group*		*Adjuvant CRT*	
	N^a^ (5‐yearOS, %)	aHR (95% CI)^b^	N^a^ (5‐yearOS, %)	aHR (95% CI)^b^	N^a^ (5‐yearOS, %)	aHR (95% CI)^b^	N^a^ (5‐yearOS, %)	aHR (95% CI)^b^
Gender								
Male	151 (27.4)	1.00	273 (29.7)	0.91 (0.70, 1.17)	134 (27.2)	0.96 (0.69, 1.34)	348 (38.0)	**0.69 (0.53, 0.88)**
Female	174 (19.9)	1.00	261 (29.5)	0.80 (0.62, 1.03)	39 (18.0)	0.81 (0.50, 1.30)	337 (31.1)	**0.73 (0.58, 0.92)**
*P‐interaction*				*0.26*		*0.89*		***<0.01***
tumor site								
Upper	69 (20.5)	1.00	144 (24.3)	1.03 (0.71, 1.49)	149 (27.7)	0.97 (0.67, 1.41)	157 (29.7)	0.89 (0.61, 1.28)
Middle	90 (20.6)	1.00	173 (35.9)	0.84 (0.59, 1.18)	9 (0)	0.92 (0.38, 2.19)	212 (42.2)	**0.58 (0.41, 0.81)**
Lower	117 (25.1)	1.00	144 (33.2)	0.77 (0.56, 1.05)	11 (0)	1.47 (0.60, 3.62)	249 (35.2)	**0.66 (0.50, 0.88)**
*P‐interaction*				*0.69*		*0.74*		***<0.01***
Stage at diagnosis								
II	120 (31.4)	1.00	171 (50.5)	0.78 (0.55, 1.09)	61 (30.7)	1.08 (0.68, 1.71)	211 (55.0)	**0.63 (0.45, 0.88)**
III	181 (13.7)	1.00	331 (19.3)	0.85 (0.69, 1.06)	106 (22.6)	0.74 (0.53, 1.02)	463 (25.8)	**0.68 (0.55, 0.83)**
*P‐interaction*				***<0.01***		***<0.01***		***<0.01***
IIIa	75 (18.5)	1.00	123 (28.3)	0.73 (0.52, 1.04)	63 (25.0)	0.83 (0.52, 1.31)	210 (30.5)	**0.68 (0.50, 0.94)**
IIIb	51 (7.3)	1.00	101 (18.1)	0.99 (0.66, 1.48)	22 (20.8)	0.52 (0.25, 1.11)	149 (26.9)	0.72 (0.49, 1.05)
IIIc	55 (12.5)	1.00	107 (7.9)	0.90 (0.59, 1.37)	21 (9.5)	0.73 (0.38, 1.42)	104 (12.9)	**0.64 (0.42, 0.95)**
Lymph nodes removed								
<16	152 (20.3)	1.00	183 (27.5)	0.78 (0.60, 1.01)	93 (22.7)	0.84 (0.59, 1.19)	281 (31.2)	**0.64 (0.50, 0.83)**
≥16 to <30	114 (24.1)	1.00	217 (29.3)	1.07 (0.79, 1.45)	60 (30.3)	0.96 (0.60, 1.53)	272 (36.6)	0.83 (0.62, 1.11)
≥30	46 (22.9)	1.00	126 (34.4)	0.64 (0.39, 1.03)	14 (27.1)	0.91 (0.40, 2.09)	126 (39.9)	0.63 (0.40, 1.01)
*P‐interaction*				*<0.01*		*<0.01*		***<0.01***

Note

Boldface indicates statistical significance (*P* < .05).

Abbreviation: aHR, adjusted harzard ratio.

^a^ N refers to the overall sample size of the corresponding row and column.

^b^ All causes of mortality was defined as the primary endpoint in the model.

**FIGURE 4:**
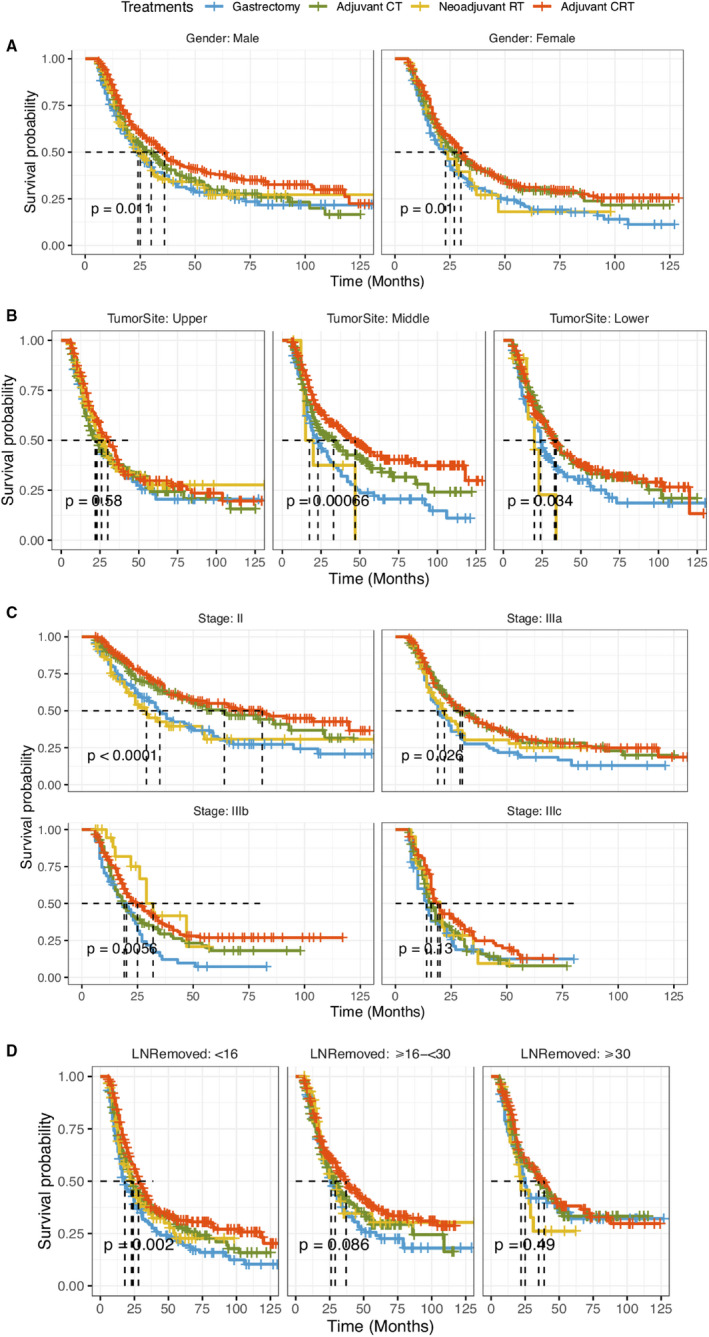
Adjusted overall survival curves for GSRC patients by (A) gender, (B) tumor site, (C) stage, (D) lymph nodes removed

Moreover, in stratified analyses of male and female GSRC patients in Figure 4A, and patients with middle tumor site, lower tumor site in Figure 4B, and subset analyses excluding stage IIIc in Figure 4C, as well as in these same analyses limited to patients with <16 lymph nodes removed in Figure 4D, patients with adjuvant CRT continued to demonstrate significantly increased overall survival rates compared to patients received gastrectomy alone.

## 4 DISCUSSION

Using SEER 2006‐2016 database, we found that patients with primary stage II‐III GSRC selected to receive CRT had improved overall survival compared to those received gastrectomy alone, which suggests that the patients of locally advanced GSRC with a recognized poor prognosis can benefit from CRT. While adjuvant CT was also positively associated with improving survival in a univariate logistic regression, the effect was not significant in the multivariable logistic regression. No positive effect was found for neoadjuvant RT group. The effect of adjuvant CRT kept stable in different gender and stage at diagnosis, and the measure turned null in none lymph nodes removed and upper tumor site like gastroesophageal junction subset.

The adjuvant CRT remains a recommended treatment therapy for gastric cancer,^22^, ^23^ but the results was limited and not consistent when the effect comes to GSRC. Our study confirmed the previous study of 1889 patients with diffuse‐type gastric cancer in stages IB‐IV, including 1454 GSRC patients between 2002 and 2005, as reported by American radiation oncologist Alexander. The median survival time was 30 months in the adjuvant CRT group vs 18 months in the non‐CRT group (*P* < .001) with the improvement in OS (HR = 0.75, *P* < .001).^24^ Given the improvements in medical techniques and clinical treatment in the recent decades, our study further verifies the conclusion using a more recent data. To our best knowledge, the chemoresistance and utility of perioperative chemotherapy for GSRC is disputed. Voron et al reported that the administration of postoperative chemotherapy had protective trend (HR = 0.873, 95% CI: 0.708, 1.077), however, did not independently influence survival in the 899 GSRC patients.^25^ As the same, Wei et al reported that postoperative chemotherapy did not improve survival (HR = 0.935, 95% CI: 0.674, 1.296) in 859 stage II‐III GSRC patients.^26^ Another study from Shi et al showed that survival benefits (HR = 0.28, 95% CI: 0.24, 0.32) of postoperative chemotherapy in 2815 stage IV GSRC patients.^27^ Wei's study reported that postoperative radiotherapy can provide a better survival in locally advanced GSRC patients (HR = 0.788, 95% CI: 0.64, 0.94).^26^ This result provides the evidence for application of adjuvant RT on treatment of GSRC patients. In our study, we offer an alternative and effective treatment of adjuvant CRT for GSRC patients by analyzing the recent nationwide survey data.

We have some speculations about possible underlying mechanisms behind the association patterns in our analysis. Firstly, adjuvant CRT can provide a locoregional control on positive lymph nodes invasion, which will contribute to the patients’ improving survival.^22^, ^24^, ^28^ The Korean ARTIST trial reported a significant advantage of adjuvant CRT on DFS in patients by treating on pathologically positive lymph nodes (*P* = .0365).^29^ Second, adjuvant CRT may provide prophylactic radiation to control potentially metastatic lymphnodes belonging to the next station.^30^ In AJCC staging system, nodal stage is based on the number of lymph nodes removed, which does not authentically reflect regularity of lymph nodes metastasis. Therefore, attention should be paid to the control of potentially metastatic lymph nodes. Third, incomplete resections (R1) are more common in GSRC^31^ and the adjuvant CRT can probably decrease the risk of local regional recurrence, especially in residual stomach and excision margins on the tumor side, ie the anastomosis, to improve survival of patients who underwent R1 resection.^28^ Dikken et al reported that adjuvant CRT significantly improved survival after R1 resections (66% vs 29%, *P* < .002). Furthermore, adjuvant CRT showed the benefit in local recurrence rate in R0 resection group (5% vs 13%, *P* < .03).^32^

### 4.1 Strengths and limitations

This is the first large‐scale population‐base study focused on treatment strategies of GSRC with a 10‐year follow‐up time span. Our study highlights the big effect of adjuvant CRT on GSRC patients’ survival. It provides a further strong evidence and inspiration on tailored treatment strategy of GSRC. This study also analyzed the effect of CMT on survival in multidimensions with robust statistics such as univariate analysis, multivariable Cox proportional hazards model and different subgroups, that could greatly diminish the impact of confounders and explore potential effect in certain group. Furthermore, we specify the time‐window of GSRC patients by excluding those who survived less than half a year to avoid the impact from patients’ poor physical health condition and adverse effects of treatment and reflect the effect of CMT accurately and convincingly.

Although this study has such strengths above, it still has several limitations. First, although the multivariable analysis adjusted for measured covariates, we were unable to control for unreported prognosis factors, such as lymphatic invasion, vascular invasion, tumor biomarkers, chemotherapy regimens, and radiotherapy regimens. Because of the lack of information on treatment cycles and dose, it is possible that patients did not complete the full cycles of CRT or received nonstandard regimen. Second, in order to examine the long‐term effects of CMT, we restrict the participants which will result in selection bias. Third, the standardization of pathological definitions for GSRC is changing in different version of the WHO classification; thus, the GSRC type may have included a small percentage of intestinal type lead to a survival benefit of CRT. Our current study does not report the proportions of signet ring cell in GSRC. Lastly, SEER did not collect information regarding gastrectomy surgical margin status (R0 resection rate), which is a significant indicator of GSRC prognosis. Thus, we were not able to further evaluate the effect of different gastrectomy methods, such as proximal, distal, subtotal, or total gastrectomy.

## 5 CONCLUSION

This study suggests that the locally advanced GSRC patients will benefit from the use of adjuvant CRT technique and a group of patients (eg middle/lower tumor site or have lymph nodes removed) can be candidate for CRT. More relevant researches should be encouraged to explore the most appropriate treatment strategy for GSRC patients for a better long‐term prognosis.

## 6 ETHICS APPROVAL AND CONSENT TO PARTICIPATE

As the data used was from SEER dataset (public). Ethics approval and consent to participate could be checked in SEER.

## CONFLICT OF INTEREST

None reported.

## AUTHOR CONTRIBUTIONS

Yang Li: Conceptualization, data curation, methodology, formal analysis, visualization, writing‐original draft, and writing‐review and editing. Zhikai Zhu: Conceptualization, data curation, methodology, formal analysis, visualization, writing‐original draft, and writing‐review and editing. Fuhai Ma: Conceptualization, writing‐review and editing. LiyanXue: Conceptualization, writing‐review and editing. Yantao Tian: Conceptualization, methodology, writing‐review and editing, project administration and supervision.

## Supporting information

Fig S1

## Data Availability

All data included in this study are available on reasonable request from the corresponding author.

## References

[1] Bray F , Ferlay J , Soerjomataram I , Siegel RL , Torre LA , Jemal A . Global cancer statistics 2018: GLOBOCAN estimates of incidence and mortality worldwide for 36 cancers in 185 countries. CA Cancer J Clin. 2018;68(6):394‐424. 30207593 10.3322/caac.21492

[2] Hu B , El Hajj N , Sittler S , Lammert N , Barnes R , Meloni‐Ehrig A . Gastric cancer: classification, histology and application of molecular pathology. J Gastrointest Oncol. 2012;3(3):251‐261. 22943016 10.3978/j.issn.2078-6891.2012.021 PMC3418539

[3] Mariette C , Carneiro F , Grabsch HI , et al. Consensus on the pathological definition and classification of poorly cohesive gastric carcinoma. Gastric Cancer. 2019;22(1):1‐9. 30167905 10.1007/s10120-018-0868-0

[4] Lauwers GCF , Graham D , Curado M , et al. Gastric carcinoma. WHO classification of tumors of the digestive system In: Bosman FCF, Hruban R, Theise N, eds. Lyon: JARC; 2010:48‐58.

[5] Henson DE , Dittus C , Younes M , Nguyen H , Albores‐Saavedra J . Differential trends in the intestinal and diffuse types of gastric carcinoma in the United States, 1973–2000 Increase in the Signet Ring Cell Type. Arch Pathol Lab Med. 2004;128(7):765‐770. 15214826 10.5858/2004-128-765-DTITIA

[6] Pokala SK , Zhang C , Chen Z , et al. Incidence, survival, and predictors of lymph node involvement in early‐stage gastric signet ring cell carcinoma in the US. J Gastrointest Surg. 2018;22(4):569‐577. 29313289 10.1007/s11605-017-3500-4

[7] Pernot S , Voron T , Perkins G , Lagorce‐Pages C , Berger A , Taieb J . Signet‐ring cell carcinoma of the stomach: Impact on prognosis and specific therapeutic challenge. World J Gastroenterol. 2015;21(40):11428‐11438. 26523107 10.3748/wjg.v21.i40.11428 PMC4616218

[8] Japanese Gastric Cancer Association . Japanese gastric cancer treatment guidelines 2014 (ver. 4). Gastric Cancer. 2017;20(1):1‐19. 10.1007/s10120-016-0622-4 PMC5215069 27342689

[9] Charalampakis N , Nogueras González GM , Elimova E , et al. The proportion of signet ring cell component in patients with localized gastric adenocarcinoma correlates with the degree of response to pre‐operative chemoradiation. Oncology. 2016;90(5):239‐247. 27046280 10.1159/000443506 PMC4870109

[10] Messager M , Lefevre JH , Pichot‐Delahaye V , et al. The impact of perioperative chemotherapy on survival in patients with gastric signet ring cell adenocarcinoma: a multicenter comparative study. Ann Surg. 2011;254(5):pp. 684–93; discussion 93. 10.1097/SLA.0b013e3182352647 22005144

[11] Bekkar S , Gronnier C , Messager M , et al. The impact of preoperative radiochemotherapy on survival in advanced esophagogastric junction signet ring cell adenocarcinoma. Ann Thorac Surg. 2014;97(1):303‐310. 24200394 10.1016/j.athoracsur.2013.09.010

[12] Heger U , Sisic L , Nienhüser H , et al. Neoadjuvant therapy improves outcomes in locally advanced signet‐ring‐cell containing esophagogastric adenocarcinomas. Ann Surg Oncol. 2018;25(8):2418‐2427. 29855828 10.1245/s10434-018-6541-3

[13] Doll KM , Rademaker A , Sosa JA . practical guide to surgical data sets: Surveillance, Epidemiology, and End Results (SEER) database. JAMA Surg. 2018;153(6):588‐589. 29617544 10.1001/jamasurg.2018.0501

[14] Surveillance, Epidemiology, and End Results (SEER) Program (www.seer.cancer.gov) SEER*Stat Database: Incidence ‐ SEER 9 Regs Research Data, Nov 2018 Sub (1975‐2016) <Katrina/Rita Population Adjustment> ‐ Linked To County Attributes ‐ Total U.S., 1969‐2017 Counties, National Cancer Institute, DCCPS, Surveillance Research Program, released April 2019, based on the November 2018 submission. [Internet]

[15] Wang X , Zhao L , Liu H , et al. A phase II study of a modified FOLFOX6 regimen as neoadjuvant chemotherapy for locally advanced gastric cancer. Br J Cancer. 2016;114(12):1326‐1333. 27172250 10.1038/bjc.2016.126 PMC4984457

[16] Edge SB , Compton CC . The American Joint Committee on Cancer: the 7th Edition of the AJCC Cancer Staging Manual and the Future of TNM. Ann Surg Oncol. 2010;17(6):1471‐1474.. 20180029 10.1245/s10434-010-0985-4

[17] Zhou R , Yan S , Li J . Influence of marital status on the survival of patients with gastric cancer. J Gastroenterol Hepatol. 2016;31(4):768‐775. 26513206 10.1111/jgh.13217

[18] American Joint Committee on Cancer . AJCC Cancer Staging Manual, 8th edn. Heidelberg: Springer; 2017.

[19] Liu Y‐Y , Fang W‐L , Wang F , et al. Does a higher cutoff value of lymph node retrieval substantially improve survival in patients with advanced gastric cancer?‐Time to embrace a new digit. Oncologist. 2017;22(1):97‐106. 27789777 10.1634/theoncologist.2016-0239 PMC5313276

[20] Schlesinger‐Raab A , Mihaljevic AL , Egert S , et al. Outcome of gastric cancer in the elderly: a population‐based evaluation of the Munich Cancer Registry. Gastric Cancer. 2016;19(3):713‐722. 26260874 10.1007/s10120-015-0527-7

[21] Chon HJ , Hyung WJ , Kim C , et al. Differential prognostic implications of gastric signet ring cell carcinoma: stage adjusted analysis from a single high‐volume center in Asia. Ann Surg. 2017;265(5):946‐953. 27232252 10.1097/SLA.0000000000001793 PMC5389602

[22] Smalley SR , Benedetti JK , Haller DG , et al. Updated analysis of SWOG‐directed intergroup study 0116: a phase III trial of adjuvant radiochemotherapy versus observation after curative gastric cancer resection. J Clin Oncol. 2012;30(19):2327‐2333. 22585691 10.1200/JCO.2011.36.7136 PMC4517071

[23] Macdonald JS , Smalley SR , Benedetti J , et al. Chemoradiotherapy after surgery compared with surgery alone for adenocarcinoma of the stomach or gastroesophageal junction. N Engl J Med. 2001;345(10):725‐730. 11547741 10.1056/NEJMoa010187

[24] Stessin AM , Sison C , Schwartz A , Ng J , Chao CK , Li B . Does adjuvant radiotherapy benefit patients with diffuse‐type gastric cancer? Results from the Surveillance, Epidemiology, and End Results database. Cancer. 2014;120(22):3562‐3568. 25043858 10.1002/cncr.28913

[25] Voron T , Messager M , Duhamel A , et al. Is signet‐ring cell carcinoma a specific entity among gastric cancers? Gastric Cancer. 2016;19(4):1027‐1040. 26606931 10.1007/s10120-015-0564-2

[26] Wei F , Lyu H , Wang S , Chu Y , Chen F . Postoperative radiotherapy improves survival in gastric signet‐ring cell carcinoma: a SEER database analysis. J Gastric Cancer. 2019;19(4):393‐407. 31897342 10.5230/jgc.2019.19.e36 PMC6928086

[27] Shi T , Song X , Liu Q , et al. Survival benefit of palliative gastrectomy followed by chemotherapy in stage IV gastric signet ring cell carcinoma patients: a large population‐based study. Cancer Med. 2019;8(13):6010‐6020. 31448584 10.1002/cam4.2521 PMC6792481

[28] Yu JI , Lim DH , Ahn YC , et al. Effects of adjuvant radiotherapy on completely resected gastric cancer: a radiation oncologist's view of the ARTIST randomized phase III trial. Radiother Oncol. 2015;117(1):171‐177. 26299196 10.1016/j.radonc.2015.08.009

[29] Lee J , Lim DH , Kim S , et al. Phase III trial comparing capecitabine plus cisplatin versus capecitabine plus cisplatin with concurrent capecitabine radiotherapy in completely resected gastric cancer with D2 lymph node dissection: the ARTIST trial. J Clin Oncol. 2012;30(3):268‐273. 22184384 10.1200/JCO.2011.39.1953

[30] Haijun YU , Qiuji WU , Zhenming FU , et al. A new approach to delineating lymph node target volumes for post‐operative radiotherapy in gastric cancer: A phase II trial. Radiother Oncol. 2015;116(2):245‐251. 26228969 10.1016/j.radonc.2015.07.010

[31] Piessen G , Messager M , Leteurtre E , Jean‐Pierre T , Mariette C . Signet ring cell histology is an independent predictor of poor prognosis in gastric adenocarcinoma regardless of tumoral clinical presentation. Ann Surg. 2009;250(6):878‐887. 19855261 10.1097/SLA.0b013e3181b21c7b

[32] Dikken JL , Jansen EPM , Cats A , et al. Impact of the extent of surgery and postoperative chemoradiotherapy on recurrence patterns in gastric cancer. J Clin Oncol. 2010;28(14):2430‐2436. 20368551 10.1200/JCO.2009.26.9654

