# Supplementary figures and images for: Improving survival of stage II‐III primary gastric signet ring cell carcinoma by adjuvant chemoradiotherapy
Source: Cancer Med. 2020 Aug 3;9(18):6617–28. doi: 10.1002/cam4.3342 (PMC7520351; doi:10.1002/cam4.3342)

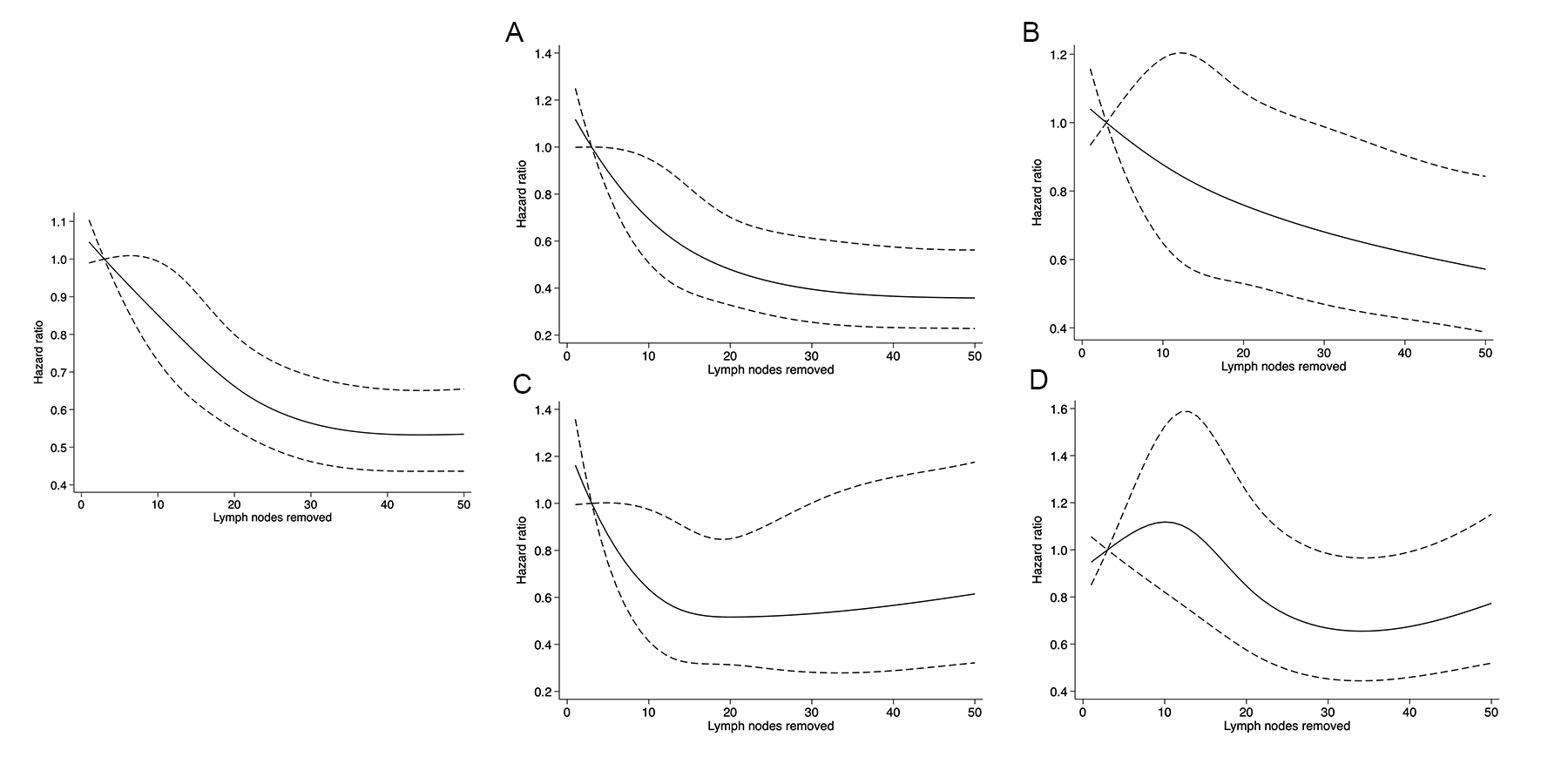

Supplement: Supplementary file 1 — Fig S1 [file CAM4-9-6617-s001.png]
